# Bayesian model evidence as a practical alternative to deviance information criterion

**DOI:** 10.1098/rsos.171519

**Published:** 2018-03-21

**Authors:** C. M. Pooley, G. Marion

**Affiliations:** 1 The Roslin Institute, The University of Edinburgh, Midlothian EH25 9RG, UK; 2 Biomathematics and Statistics Scotland, James Clerk Maxwell Building, The King's Buildings, Peter Guthrie Tait Road, Edinburgh EH9 3FD, UK

**Keywords:** Bayes' factor, Bayesian model evidence, marginal likelihood, Markov chain Monte Carlo, thermodynamic integration, deviance information criterion

## Abstract

While model evidence is considered by Bayesian statisticians as a gold standard for model selection (the ratio in model evidence between two models giving the Bayes factor), its calculation is often viewed as too computationally demanding for many applications. By contrast, the widely used deviance information criterion (DIC), a different measure that balances model accuracy against complexity, is commonly considered a much faster alternative. However, recent advances in computational tools for efficient multi-temperature Markov chain Monte Carlo algorithms, such as steppingstone sampling (SS) and thermodynamic integration schemes, enable efficient calculation of the Bayesian model evidence. This paper compares both the capability (i.e. ability to select the true model) and speed (i.e. CPU time to achieve a given accuracy) of DIC with model evidence calculated using SS. Three important model classes are considered: linear regression models, mixed models and compartmental models widely used in epidemiology. While DIC was found to correctly identify the true model when applied to linear regression models, it led to incorrect model choice in the other two cases. On the other hand, model evidence led to correct model choice in all cases considered. Importantly, and perhaps surprisingly, DIC and model evidence were found to run at similar computational speeds, a result reinforced by analytically derived expressions.

## 1. Introduction

The advent of Markov chain Monte Carlo (MCMC) has enabled Bayesian inference for increasingly complex models [1], but much of this progress has been in the context of single model inference. Model selection refers to the problem of selecting or weighting different models in the light of the available data. One of the key challenges is to avoid overfitting the data by selecting unduly complex models. More reliable and efficient model selection is critical to the wider adoption of model-based scientific discovery, especially for applications in dynamic process-based modelling. Multi-model Bayesian inference automatically, and implicitly, includes a penalty for unnecessary model complexity, and thus guards against overfitting [2,3]. Reversible jump MCMC, introduced by Green [4], in principle can be used to implement Bayesian model choice (this estimates the model posterior probability by the proportion of samples the MCMC chain spends within that model). However, this approach can be difficult to implement in practice [5].

From a Bayesian perspective, deviance information criterion (DIC) is an approximate model selection method which tries to explicitly balance model complexity with fit to the data [6]. However, there are increasing concerns with regard to its discriminatory performance [7], particularly in the presence of latent variables where there is no unique definition [8]. The practical issues that arise in the implementation of fully Bayesian approaches to model choice are illustrated by the fact that, for example, DIC is the only model selection tool in widespread use for assessing the fit of dynamic stochastic epidemiological models [9–11]. The aim of this paper is to point out the potential unreliability of DIC and to show that Bayesian model selection can be undertaken in a statistically consistent way that is not hard to implement and not substantially computationally slower (and is actually faster in some cases).

We focus on stochastic models that contain parameters *θ* and latent variables *x.* To take an epidemiological example, the parameters describe the dynamics of the system (e.g. the average infection and recovery rates of individuals) and the latent variables are the unobserved consequences of those dynamics (e.g. the infection and recovery events). The model latent space behaviour is characterized by a ‘latent process likelihood’ *π*(*x*|*θ*). We assume that an ‘observed data likelihood’ *P*(*y*|*θ*,*x*) describes the probability of some observed data *y*, given a particular set of latent variables *x* and model parameters *θ*.^1^ If we define a prior distribution *π*(*θ*), then Bayes' theorem enables the posterior distribution to be expressed as

1.1 $$P(\theta ,x\;|\;y)=\frac{P(y\;|\;\theta ,x)\pi (x\;|\;\theta )\pi (\theta )}{P(y)}\;,$$

where the normalizing factor

1.2 P(y)=∫P(y | θ,x)π(x | θ)π(θ) dθ dx

is known as the model evidence, or marginal likelihood [12]. Note, in the above equation, the dependence on the particular model *m* is implicit. When the prior belief ascribed to each model is equal, this measure forms the sole basis of Bayesian model selection, i.e. the ranking of competing models in the light of the data, with the highest evidence identifying the best model. The famous Bayes’ factor [13] comparing models *m*_1_ and *m*_2_ is simply the ratio of the evidence given by data *y* to model *m*_1_ relative to that given by model *m*_2_:

1.3 $$B_{1,2}=\frac{P(y\;|\;m_{1})}{P(y\;|\;m_{2})}.$$

A Bayes factor of 10 is typically considered to represent strong evidence favouring *m*_1_ over *m*_2_ [14].

Recently, a variety of techniques have been developed for calculating the model evidence either exactly, e.g. through annealed importance sampling (AIS) [15] or steppingstone sampling (SS) [16], or approximately, e.g. using thermodynamic integration [17–20]. These approaches enable evidence calculation one model at a time and thus represent a practical alternative to reversible jump MCMC for Bayesian model selection.^2^ This paper uses a version of SS, as described in the next section. Section 3 introduces DIC and its relationship to model evidence and §4 presents analytical estimates for the computational efficiency of the two approaches. In §5, both the accuracy and efficiency of DIC and SS are assessed using three benchmark models: linear regression models, mixed models (which contain latent random effects) and Markovian stochastic compartmental models widely used to describe epidemic processes. Finally, conclusions are drawn in §6.

## 2. Model evidence using steppingstone sampling

SS, introduced by Xie *et al*. [16], calculates the model evidence by means of generating samples from *K* separate MCMC chains at different inverse temperatures *ϕ*_1_ = 1 > *ϕ*_2_ > … > *ϕ_K_* = 0. Each chain undergoes changes as a result of proposals that are either accepted or rejected. These proposals can take a variety of forms (e.g. random walk [1], Gibbs sampling [21], Metropolis-adjusted Langevin algorithm [22] etc.) and must be selected such that the MCMC chain can, in principle at least, explore the entirety of parameter and latent variable space. A key difference between SS and ordinary MCMC is that the proposals for each chain *k* use a Metropolis–Hastings acceptance probability modified by the chain's inverse temperature *ϕ_k_*:

2.1 $$\left\{{\left(\frac{P(y\;|\;\theta_{p},x_{p})}{P(y\;|\;\theta_{i}^{k},x_{i}^{k})}\right)}^{\phi_{k}}\frac{\pi (x_{p}\;|\;\theta_{p})\pi (\theta_{p})}{\pi (x_{i}^{k}\;|\;\theta_{i}^{k})\pi (\theta_{i}^{k})}\frac{j_{p\to i}}{j_{i\to p}},1\right\},$$

where $\theta_{i}^{k},x_{i}^{k}$ represents the current sample (indexed by *i*) and $\theta_{p},x_{p}$ represents a proposal (with probability $j_{i\to p}$). If the proposal is accepted then the next sample on the chain is set to $\theta_{i+1}^{k}=\theta_{p},x_{i+1}^{k}=x_{p}$, otherwise $\theta_{i+1}^{k}=\theta_{i}^{k},x_{i+1}^{k}=x_{i}^{k}$. Repeated application of equation (2.1) on each of the chains generates samples distributed in proportion to

2.2 $$P{\left(y\;|\;\theta ,x\right)}^{\phi_{k}}\pi (x\;|\;\theta )\pi (\theta ).$$

Consequently, the chain *ϕ*_1_ = 1 samples from the posterior (see equation (1.1)), *ϕ_K_* = 0 samples from the prior^3^ and chains in between these two extremes provide steppingstones going from one to the other. An unbiased approximation to the model evidence is then given by [16]

2.3 $$\hat{P}(y)=\prod_{k=2}^{K}\left(\frac{1}{N}\sum_{i=1}^{N}P{\left(y\;|\;\theta_{i}^{k},x_{i}^{k}\right)}^{{\;}^{\phi_{k-1}-\phi_{k}}}\right),$$

which converges on the true model evidence as the number of samples *N* tends to infinity (see electronic supplementary material, appendix A, for a derivation of this expression).

**Figure 1.**
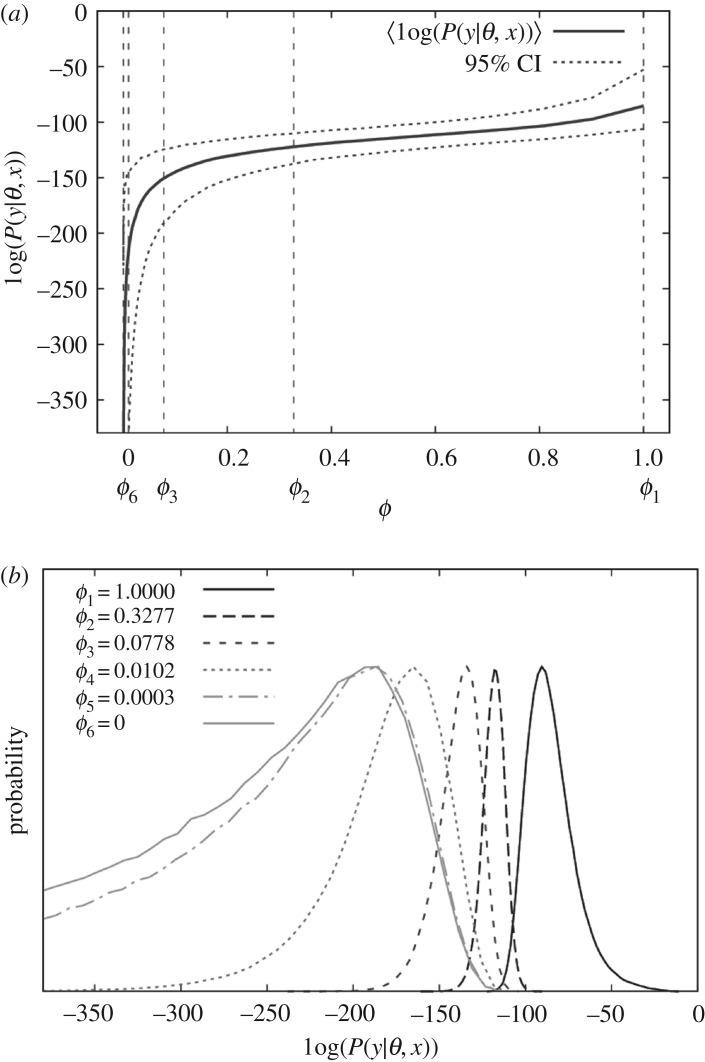
(*a*) A typical example of how the posterior distribution in the log of the observed data likelihood varies as a function of inverse temperature *ϕ*. This distribution is represented by a mean (solid line) and 95% confidence intervals (denoted by the dotted lines). (*b*) The distributions for *K *= 6 chains from which samples are drawn during SS (with inverse temperatures defined by equation (2.4)).

SS can be illustrated by means of figure 1, which shows *K *= 6 chains. The solid line in figure 1*a* shows the variation in the mean of the log of the observed data likelihood as a function of inverse temperature. Note, it decreases from right (posterior) to left (prior), as would be expected. The vertical dashed lines in this diagram represent the inverse temperatures of the different chains which are chosen in accordance with

2.4 $$\phi_{k}={\left(\frac{K-k}{K-1}\right)}^{5}.$$

This commonly used power-law scaling is suboptimal (a better but more complicated adaptive scheme is implemented in Friel *et al*. [19]), but empirically is found to work for a large number of problems. By design, it focuses most chains at low inverse temperatures where changes in the observed data likelihood (and variance) are greatest. Figure 1*b* shows the distributions from which samples are generated. As will be discussed later, algorithm efficiency is relatively insensitive to *K*, provided *K* is sufficiently large to allow for a substantial overlap between distributions from adjacent chains. In this study, *K* is chosen to be 50 to ensure this condition is satisfied for all cases investigated. Note even though 50 chains are being updated instead of just one for DIC, it does not imply that the SS method is 50 times slower. This is because the estimate for the model evidence in equation (2.3) converges more rapidly than the corresponding DIC measures, and so, fewer MCMC iterations are required to obtain a given accuracy (as shown in §4).

This paper runs an implementation of SS in which the *K* chains are updated in parallel with swapping of states between adjacent chains to improve mixing (for details, see electronic supplementary material, appendix A). It should be noted, however, that in some applications, finite memory restrictions make running a large number of chains problematic. In such cases, a scanning approach may be adopted where samples are generated in a sequential manner.^4^

## 3. Deviance information criterion

Within classical model fitting, the problem of model selection is achieved by considering two competing notions: firstly, a measure of model fit that promotes selecting more accurate models and, secondly, a measure of model complexity, often represented by the number of parameters *p_m_* in the model. This penalty discourages overfitting (increasing *p_m_* almost always improves goodness of fit). A commonly used measure that balances these two contributions is Akaike's information criterion (AIC) [23]:

3.1 $$\text{AIC}=-2\log(P(y\;|\;\theta_{\max}))+2p_{m},$$

where *θ*_max_ is the parameter set that maximizes the observed data likelihood *P*(*y*|*θ*). Given a number of candidate models, the one with the smallest AIC value is considered best.

For complex hierarchical models, however, establishing *p_m_* is problematic due to a lack of independence between parameters. This has led to the introduction of DIC which uses MCMC results directly. In contrast with SS, however, DIC uses samples from a single posterior chain *k* = 1. As shown below, there are actually multiple definitions for DIC in the literature.

### 3.1. Deviance information criterion for problems without latent variables

Considering models that do not contain latent variables *x*, two contrasting definitions for DIC have been proposed. Firstly, Spiegelhalter *et al.* [6] suggested

3.2 $$\text{DI}{\text{C}}_{1}=-2\langle \log(P(y\;|\;\theta ))\rangle +2(log(P(y\;|\;\langle \theta \rangle ))-\langle \log(P(y\;|\;\theta ))\rangle ),$$

where 〈…〉 denotes an average over the posterior distribution (note, with a rearrangement, this expression is analogous to AIC in equation (3.1) if we associate log(*P*(*y*|〈*θ*〉)) with log(*P*(*y*|*θ*_max_)) and 2[〈log(*P*(*y*|*θ*)〉 − log(*P*(*y*|〈*θ*〉))] with effective parameter number *p_m_*). Secondly, Gelman [24] proposed

3.3 $$\text{DI}{\text{C}}_{2}=-2\langle \log(P(y\;|\;\theta ))\rangle +2\;\text{var}[\log(P(y\;|\;\theta ))],$$

where now the effective parameter number is given by twice the posterior variance of the observed data likelihood.

### 3.2. Deviance information criterion for problems with latent variables

Defining a DIC measure in cases when the model contains latent variables is problematic (for example, Celeux *et al.* [8] investigated eight potential definitions). Analogous to equations (3.2) and (3.3), one possibility is simply to use the observed data likelihood as before, but now average over the latent space *x:*

3.4 $$\begin{array}{ll} & {\text{DIC}}_{3}=-2\langle \log(P(y\;|\;\theta ,x))\rangle +2(\langle \log{(P(y\;|\;{\langle \theta \rangle }_{\theta |x},x)\rangle }_{x}-\langle \log(P(y\;|\;\theta ,x))\rangle )\\ \text{and} & {\text{DIC}}_{4}=-2\langle \log(P(y\;|\;\theta ,x))\rangle +2\text{ var}[\log(P(y\;|\;\theta ,x))]. \end{array}\}$$

Here, 〈…〉 represents an average over the full posterior, 〈…〉*_θ_*_|_*_x_* is the posterior average over parameters *θ*, given a particular latent variable state *x*, and 〈…〉*_x_* a posterior average over the latent variable space. A second option considered by Celeux *et al.* [8] is to use the complete posterior probability (i.e. including the contribution from the latent process likelihood as well as the observed data likelihood):

3.5 $$\begin{array}{rl} \text{DI}{\text{C}}_{5} & =-2\langle \log(P(y,x\;|\;\theta ))\rangle +2({\left\langle \log(P(y,x\;|\;{\left\langle \theta \right\rangle }_{\theta \;|\;x}))\right\rangle }_{x}-\langle \log(P(y,x\;|\;\theta ))\rangle )\\ \;\text{and}\;\text{DI}{\text{C}}_{6} & =-2\langle \log(P(y,x\;|\;\theta ))\rangle +2\;\text{var}[\log(P(y,x\;|\;\theta ))]. \end{array}\}$$

There is no consensus on a theoretical justification for a preference between these various options.

### 3.3. Relationship between deviance information criterion and model evidence

To investigate the relationship between model evidence and DIC, we consider a simple case for which analytical results are derivable (note, understanding this section is not necessary for the rest of the paper, so it may be skipped). Here, we assume a model with no latent variables *x* and a multi-variate normal (MVN) distribution for the observed data likelihood^5^

3.6 $$P(y\;|\;\theta ,x)=P(y\;|\;\theta )=P(y\;|\;\langle \theta \rangle )\;{\text{e}}^{-1/2{\left(\theta -\langle \theta \rangle \right)}^{T}\Sigma^{-1}(\theta -\langle \theta \rangle )},$$

where *θ* is the focus of inference, 〈*θ*〉 represents the parameter set corresponding to the maximum observed data likelihood and Σ is a covariance matrix between model parameters, both assumed known. Calculating model evidence requires a proper prior.^6^ For simplicity, we choose this to also be MVN and centred on $\bar{\theta }$:

3.7 $$\pi (\theta )=\frac{1}{\sqrt{{\left(2\pi \right)}^{d}\;|\;\Omega \;|\;}}{\text{e}}^{-1/2{\left(\theta -\bar{\theta }\right)}^{T}\Omega^{-1}(\theta -\bar{\theta })}.$$

The product of two MVNs is also MVN, so equations (3.6) and (3.7) can be multiplied and the parameters integrated out to give (see electronic supplementary material, appendix D for details)

3.8 $$P(y)=P(y\;|\;\langle \theta \rangle )\sqrt{\frac{\;|\;\psi \;|\;}{\;|\;\Omega \;|\;}}{\text{e}}^{-1/2({\left\langle \theta \right\rangle }^{T}\Sigma^{-1}\langle \theta \rangle +{\bar{\theta }}^{T}\Omega^{-1}\bar{\theta }-\mu^{T}\psi^{-1}\mu )},$$

where *μ* and *ψ* are the mean and covariance matrix of the posterior. To make a comparison with DIC, it is of use to take minus two times the log of the evidence

3.9 $$-2\log(P(y))=-2\log(P(y\;|\;\langle \theta \rangle ))+{\left\langle \theta \right\rangle }^{T}\Sigma^{-1}\langle \theta \rangle +{\bar{\theta }}^{T}\Omega^{-1}\bar{\theta }-\mu^{T}\psi^{-1}\mu -\log\left(\frac{\;|\;\psi \;|\;}{\;|\;\Omega \;|\;}\right).$$

Furthermore, calculating the posterior mean and variance of the log of the observed data likelihood in equation (3.6) and substituting them into the definitions for DIC in equations (3.2) and (3.3) yields the following analytical results

3.10 $$\begin{array}{r} \begin{array}{rl} \text{DI}{\text{C}}_{1} & =-2\log(P(y\;|\;\langle \theta \rangle ))+2[\text{Tr}(\Sigma^{-1}\psi )+\Delta \theta^{T}\Sigma^{-1}\Delta \theta ]\\ \;\text{and}\;\text{DI}{\text{C}}_{2} & =-2\log(P(y\;|\;\langle \theta \rangle ))+\text{Tr}(\Sigma^{-1}\psi )+\Delta \theta^{T}\Sigma^{-1}\Delta \theta +\text{Tr}(\Sigma^{-1}\psi \Sigma^{-1}\psi )+2\Delta \theta^{T}\Sigma^{-1}\psi \Sigma^{-1}\Delta \theta , \end{array}\} \end{array}$$

where Tr denotes the trace of a matrix and Δ*θ *= *μ* − 〈*θ*〉 (see electronic supplementary material, appendix D for details).

Note the measures given in equations (3.9) and (3.10) all share a common first term. Thus, comparison naturally focuses on the subsequent terms. We consider analysing a model with *p_m_* = 10 parameters under the following scenario: for the observed data likelihood, the diagonal elements of Σ are set to 1 and off-diagonal elements are drawn from a uniform distribution between −0.1 and 0.1, for the prior Ω is diagonal with elements *λ*^2^, both distributions are set to have a common mean$\langle \theta \rangle =\bar{\theta }=\mu$ and we arbitrarily choose *P*(*y*|〈*θ*〉) = 1.

**Figure 2.**
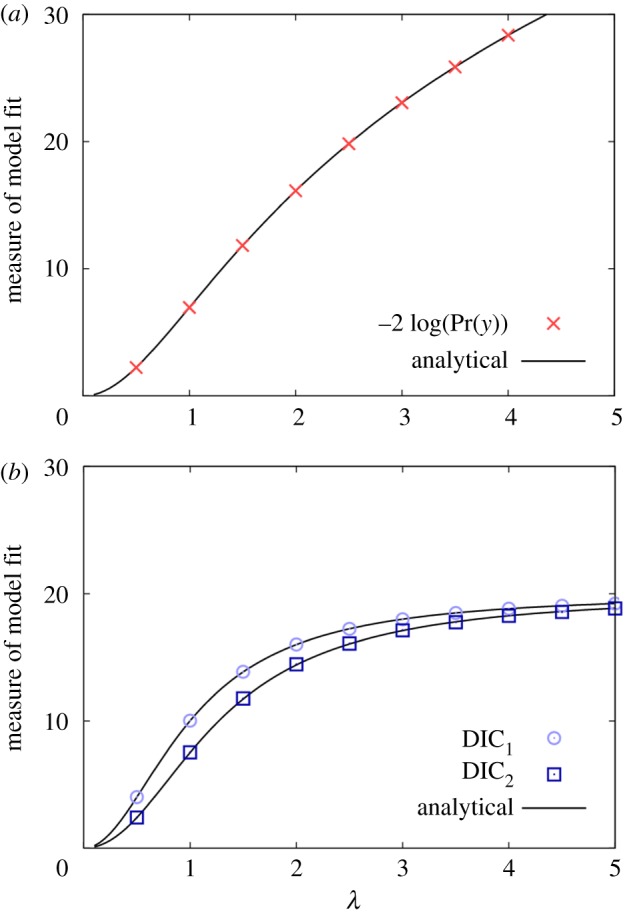
Model with MVN observed data likelihood and prior (and hence posterior). Shows how (*a*) an evidence-based model selection measure and (*b*) two DIC measures (equations (3.2) and (3.3)) vary as a function of the standard deviation in the prior distribution λ.

Figure 2 shows how the various model selection measures vary as a function of the prior standard deviation *λ*. The crosses in figure 2*a* are calculated using SS from equation (2.3) (10^3^ burn-in steps and *N* = 10^5^ iterations sufficient to generate a high degree of accuracy) and the solid black line shows that they are in excellent agreement with the analytical expression from equation (3.9). The DIC results in figure 2*b* are obtained by running a single posterior chain (10^3^ burn-in steps and *N* = 10^5^ iterations), and again agreement with the analytical results in equation (3.10) is good.

Figure 2 illustrates notable differences between DIC and evidence-based measures. It is important to emphasize that these differences do not arise from DIC inaccurately approximating the evidence. Rather, the two approaches are simply different measures arising from contrasting philosophies: (i) for model evidence, a ‘good’ model is taken to be one that when sampled from gives good agreement with the data (in this case, sampling means drawing parameters from the prior). Therefore, making the prior less and less informative (by increasing *λ*) results in agreement with the data inevitably going down, and consequently, the measure −2 log(*P*(*y*)) approaching infinity. (ii) For DIC approaches, a ‘good’ model is one that contains some set of parameters that make the data likely, and then adds in a penalty term to account for model complexity. In the limit of a flat prior, the penalty terms in equation (3.10) both tend towards 2*p_m_,* just as for AIC in equation (3.1) (hence explaining why the DIC curves in figure 2 converge on 20, given the model contains *p_m_* = 10 parameters).

It should be noted, however, that the differences in figure 2 do not validate one approach over another. This is because when performing model selection, the *absolute* value for the measure is unimportant. It is the *differences* in measure between models that are key. Therefore, validity can only be tested by investigating the ability of these approaches to correctly discriminate within a range of potential models.

Furthermore, it should be stressed that the stated aim of DIC is not to find the ‘true’ model, but rather to accurately predict future datasets in a world in which the true data generating is, in fact, very high dimensional (and effectively unobtainable) [6,7]. Nevertheless, this paper contends that in situations in which the true model *is* low dimensional and exactly know (as it is for the simulated datasets in §5), it should be expected that DIC does a reasonable job of selecting the true model, since simulation of the observed data from the true model intuitively seems more plausible than from an incorrect one. Therefore, this paper uses model selection, rather than prediction accuracy (or any other measure of model fit, such as posterior predictive assessment [25]), as a means of comparing DIC and model evidence, but concedes that this is not an entirely fair comparison.

## 4. Relative computational speed

Before assessing model selection using the evidence and DIC, we first briefly investigate the expected computational speeds with which these quantities can be accurately estimated. All the algorithms contain the same fundamental ‘update’: one in which a complete set of Metropolis–Hastings proposals are applied to allow changes in latent space (typically, this might act on each parameter and latent variable in turn). Updates take approximately the same computational time regardless of method. Therefore, one way to compare computational efficiencies is to establish how many updates *U* are required to achieve a certain level of sampling variance *ε*^2^ (i.e. noise associated with sampling error) in the corresponding model selection measure. In the case of SS, this measure is taken to be −2 log(*P*(*y*)) to allow for direct comparison with the DIC results. Assuming large *K,* the following analytical results for *U* can be derived (see electronic supplementary material, appendix E for further details):

4.1 $$\text{SS}:\frac{4}{\varepsilon^{2}}K\sum_{k=2}^{K}n_{k}^{\mathrm{cor}}\sigma_{k}^{2}{\left(\phi_{k-1}-\phi_{k}\right)}^{2}\;{\text{DIC}}_{1}:\frac{16n_{1}^{\mathrm{cor}}\sigma_{1}^{2}}{\varepsilon^{2}}\;\text{DI}{\text{C}}_{2}:\frac{8n_{1}^{\prime \mathrm{cor}}\sigma_{1}^{4}}{\varepsilon^{2}},$$

where $n_{k}^{\mathrm{cor}}$ represents the characteristic number of updates on chain *k* over which MCMC becomes uncorrelated (also represented by $n_{k}^{\prime \mathrm{cor}}$ but with a slightly different definition) and $\sigma_{k}$ is the standard deviation in the log of the observed data likelihood on chain *k* (note *k* = 1 corresponds to the posterior chain used for the DIC calculations).

The distributions for $\sigma_{k}$ and $n_{k}^{\mathrm{cor}}$ are problem-dependent, thus making it difficult to definitively say which approach is the fastest; however, the above expressions do enable a broad comparison. One key point to emphasize is that the computational efficiency for SS in equation (4.1) is *independent* of *K* for large *K* (if *K* is doubled then the temperature separation is approximately halved and these contributions cancel each other out).^7^ This result is surprising, and goes some way to explain why despite the fact that SS uses *K* = 50 chains in this study, it is comparable in speed to DIC which uses just a single chain *K* = 1.

The expressions in equation (4.1) also contain other notable features: firstly, in implementing SS, it makes sense to have smaller temperature separations at lower inverse temperatures, as this is the region in which the variance is the greatest (e.g. figure 1*b*), thus helping to motivate the temperature selection scheme in equation (2.4). Secondly, SS benefits from swapping between adjacent chains because this helps to reduce $n_{k}^{\mathrm{cor}}$. Thirdly, as the number of dimensions in the model increases (and/or the prior becomes less informative), the difference in the observed data likelihood between the prior and posterior chains will inevitably go up. This will have the consequence of increasing $\sigma_{K}$ relative to $\sigma_{1}$;^8^ hence, for very large problems, SS might be expected to be slower than DIC. Lastly, DIC_2_ contains an additional factor of $\sigma_{1}^{2}$ compared to DIC_1_. Since $\sigma_{1}^{2}$ is a measure of model complexity, this implies that DIC_2_ is expected to be slower relative to other methods as model size is increased.^9^

## 5. Assessment using benchmark models

In this section, we use simulated data to compare model selection accuracy and computational speed using the SS and DIC approaches introduced previously. Three different types of model are considered: (i) a linear regression model (which does not have latent variables and has a nearly MVN posterior distribution), (ii) a mixed model (which incorporates latent variables but again is approximately MVN), and (iii) a set of compartmental epidemic models (which are not MVN).

### 5.1. Linear regression

Consider a set of measurements *y_r_* (where *r* runs from 1 to *R*). An example would be the heights of different individuals in a population. The aim of linear regression is to help explain these measurements in terms of certain known factors, or ‘regressors’, e.g. gender, age, nationality. The model itself is described by

5.1 $$y_{r}=\sum_{j=1}^{J}X_{rj}\beta_{j}+\varepsilon_{r},$$

where *X_rj_* is a design matrix and *β_j_* are regressors (where *j* runs from 1 to *J*). For example, *β*_2_ could represent the average height difference between males and females (in which case, we might set *X_r_*_2_ = 0 for all females in the population and *X_r_*_2_ = 1 for all males). By convention, *X_r_*_1_ = 1 for all *r* such that *β*_1_ is the intercept (or average value of *y_r_* when all other regressors are set to zero). The residuals *ε_r_* in equation (5.1) account for any discrepancy between the predictions made by the regressors and the actual observations. They are assumed to be normally distributed with variance *η*^2^.

The linear regression model contains parameters *θ* = {*β*, *η*^2^} but no latent variables *x*. From equation (5.1), the observed data likelihood is given by the product of normal distributions

5.2 $$P(y\;|\;\theta ,x)=\prod_{r=1}^{R}\frac{1}{\sqrt{2\pi \eta^{2}}}{\text{e}}^{-(1/(2\eta^{2})){\left(y_{r}-\sum_{j}X_{rj}\beta_{j}\right)}^{2}}$$

and the latent process likelihood is simply *π*(*x*|*θ*) = 1.

In terms of model selection, one of the key difficulties faced by the scientist is determining which regressors are genuine (i.e. actually affect the observations) and which are not. Specifically, suppose data are available from *J′* potential regressors, where *J′* is greater than (or equal to) the true number of regressors *J*. We denote *κ* as a *J′* dimensional vector that specifies a particular model, such that *κ_j_* = 1 if the model contains regressor *j* and *κ_j_* *=* 0 otherwise. Thus, in total, $2^{J^{\prime }}$ possible models exist. If *J′* is small then it may be practical to calculate model selection measures for each possibility, but as *J′* increases, the number of potential models can become vast. In this case, two approaches can be taken: (i) in a Bayesian setting model selection MCMC [26] can be used to generate posterior samples for the vector *κ* [26] and (ii) stepwise methods [27] which minimize the model selection measure by accepting or rejecting successive changes to the model (see electronic supplementary material, appendices G and H for details).^10^

The focus of this paper, however, is not to get into the details of how model selection is actually achieved using these two approaches. Rather, a question of more fundamental importance is addressed: Is the model selection measure actually minimized at (or near to) the true model? (Note, this is an example in which it is implicit that DIC should perform well.) We address this question by considering a simple toy example. Here, *J′* = 10 regressors are assumed to exist (elements of the design matrix *X_rj_* for *j* = 2 … *J′* are set to 0 or 1 with equal probability). The first five of these regressors are assumed to actually influence the trait and the last five have no influence (i.e. the true model is represented by *κ_j_* = 1 for *j* = 1 … 5 and *κ_j_* = 0 for *j* = 6 … 10). *R* = 100 measurements are simulated from the true model by means of equation (5.1) assuming *J *= *J′*, *β_j_* = 0.5 for *j* = 1 … 5, *β_j_* = 0 for *j* = 6 … 10, and residual variance is taken to be *η*^2^ = 1.

**Figure 3.**
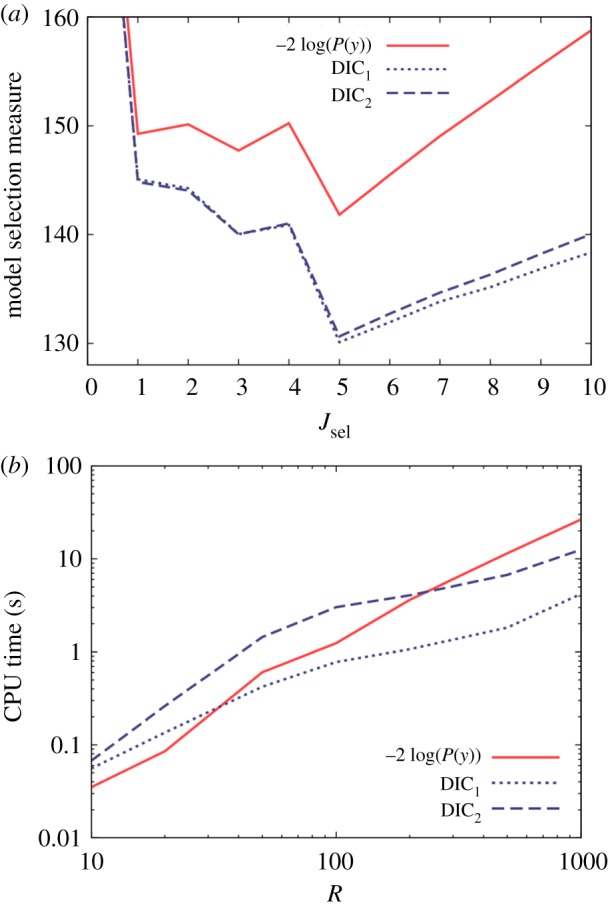
Results from a linear regression model with 10 regressors, the first five of which determine the observed data. (*a*) Model selection measures based on models with a varying number of regressors *J*_sel_, where only those regressors *J*_sel_ and below are included (hence, *J*_sel_ = 5 represents the true model). Here, *R* = 100. (*b*) CPU time necessary to accurately estimate the model selection measures (within an uncertainty of 0.2) as a function of the number of observations *R*.

Next, we consider a subset of potential models which are characterized by the number of regressors *J*_sel_ they contain and defined such that *κ_j_* = 1 for *j *≤ *J*_sel_ and *κ_j_* = 0 for *j* > *J*_sel_ (note here that *J*_sel_ = 5 represents the true model). The priors for *β_j_* are taken to be uniform between *−*2 and 2*,* and for *η*^2^ uniform between 0.1 and 2 (these bounds ensure the prior is proper and sufficiently diffuse to have a negligible effect on the posterior distribution as compared to using an infinite flat prior).^11^ Figure 3*a* shows the various model selection measures as a function of *J*_sel_. For all the results in this paper, 2 × 10^3^ burn-in updates are used, followed by *N* = 10^5^ sampling updates in the case of SS and *N* = 10^6^ updates in the case of the DIC (the details of the MCMC implementation are provided in electronic supplementary material, appendix J). The results using model evidence are shown by the solid red line. Those models with *J*_sel_ < 5 are missing certain factors that help to explain the data, so naturally they are not expected to be as good (i.e. there is less evidence supporting them, so −2 log(*P*(*y*)) is higher). Those models with *J*_sel_ > 5 contain extra model parameters not expected to provide any new information (as they were not used in generating the data). Consequently, the solid curve has a long-term upward trajectory towards the right of figure 3*a*. The minimum lies at *J*_sel_ = 5, indicating that model evidence had successfully identified the true model.

The dotted and dashed blue lines in figure 3*a* provide model selection measures using DIC from equations (3.2) and (3.3). They are both similar and in good qualitative agreement with the evidence-based results. One notable feature, however, is that DIC does not penalize against redundant parameters as strongly as the evidence (shown by the increasing separation between the blue and red curves from left to right in this diagram).

Next, we investigate the speed with which the various model selection measures can be estimated. This is achieved by running a large number (100) of independent runs of a given inference algorithm (either DIC or SS), and then calculating the CPU time after which the standard deviation in the model selection measure across runs falls below a certain critical value (which is taken to be 0.2). To remove stochastic noise, the procedure is replicated over four datasets with the CPU times being averaged (further details are given in electronic supplementary material, appendix I).

Figure 3*b* shows this CPU time as a function of the number of observations *R*.^12^ We find that DIC_2_ is consistently slower than DIC_1_, as expected from the expressions in equation (4.1), and model evidence takes a similar amount of time (despite the fact that the SS method runs using 50 chains). The curves exhibit similar scaling properties, although SS clearly becomes slower relative to DIC for larger system sizes, in line with the third point made in §4.

Illustrative annotated code (written in C++) showing the implementation of the various approaches for the linear regression model (as well as the other models below) is available in the electronic supplementary material.

### 5.2. Mixed model

In mixed models, the so-called ‘random effects’ *u_q_* (where *q* runs from 1 to *Q*) are added to equation (5.1) through a second design matrix *Z_rq_*:

5.3 $$y_{r}=\sum_{j=1}^{J}X_{rj}\beta_{j}+\sum_{q=1}^{Q}Z_{rq}u_{q}+\varepsilon_{r}.$$

These random effects are assumed to be drawn from a distribution with mean zero and covariance matrix **G**.

One application of random effects is to explain correlations in traits between genetically related individuals (in the height example used above, a person's height is, to a certain extent, related to the average height of their parents). Here, the following simplifications can be made [28]: **Z** becomes the identity matrix with *Q = R* (implying one random effect per individual)*,* and **G** = *ω*^2^**A** (where **A** is an *R* × *R* ‘relationship matrix’ that captures relatedness between individuals in the population). The relative size of genetic to residual effects (commonly termed environmental effects in this context) is captured through the heritability

5.4 $$h^{2}=\frac{\omega^{2}}{\omega^{2}+\eta^{2}}.$$

This model contains parameters *θ* = {*β*, *η*^2^, *ω*^2^} and latent variables *x* = {*u*}. From equation (5.3), the observed data likelihood is given by the product of normal distributions

5.5 $$P(y\;|\;\theta ,x)=\prod_{r=1}^{R}\frac{1}{\sqrt{2\pi \eta^{2}}}{\text{e}}^{-(1/(2\eta^{2})){\left(y_{r}-\sum_{j}X_{rj}\beta_{j}-u_{r}\right)}^{2}}$$

and the latent process likelihood is MVN

5.6 $$\pi (x\;|\;\theta )=\frac{1}{{\left(2\pi \right)}^{R/2}\omega^{R}\sqrt{\;|\;A\;|\;}}{\text{e}}^{-(1/(2\omega^{2}))u^{T}A_{}^{-1}u}.$$

To simulate data, we assume a population of 50 individuals with random mating over four generations (making *R* = 200 observations in total)*,* environmental variance *η*^2^ = 0.5 and heritability *h^2^* = 0.5. As in §5.1, we assume *J *= *J′* = 10 regressors but that only five of them actually contribute to the trait (i.e. *β_j_* = 0.5 for *j* = 1 … 5 and *β_j_* = 0 for *j* = 6 … 10). The priors for *β_j_* are taken to be uniform between −2 and 2, and for *η*^2^ and *ω*^2^ uniform between 0.1 and 2. Details of how the relationship matrix **A** is calculated are given in electronic supplementary material, appendix K, and MCMC proposals are described in electronic supplementary material, appendix L.

**Figure 4.**
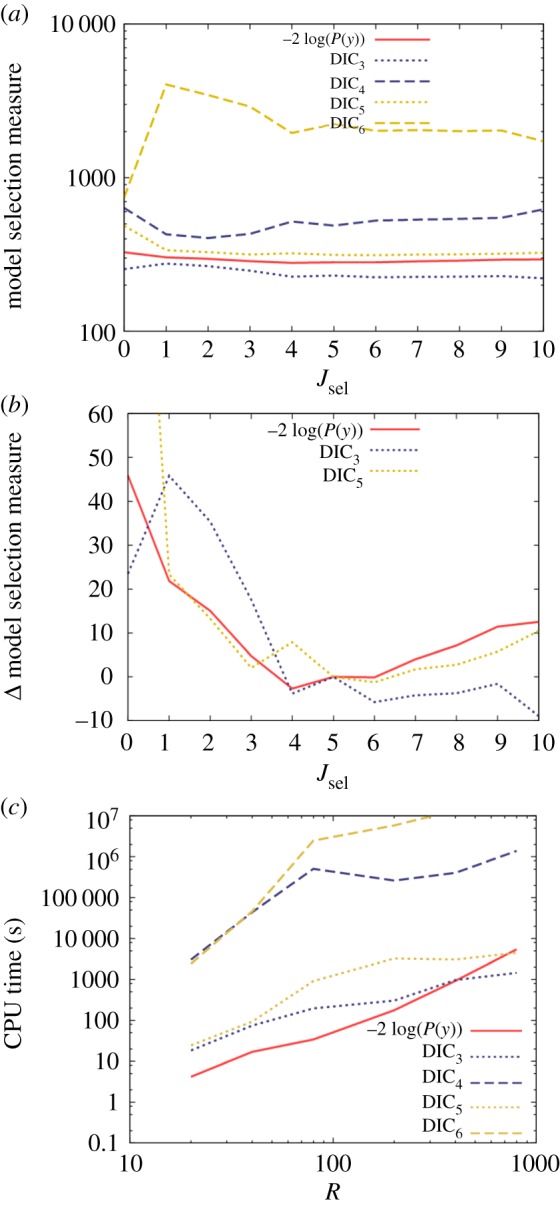
Results from a mixed model with 10 regressors (otherwise known as fixed effects), the first five of which determine the observed data, and one random effect for each individual. (*a*) Model selection measures based on models with a varying number of regressors *J*_sel_, where only those regressors *J*_sel_ and below are included (hence, *J*_sel_ = 5 represents the true model). Here, *R* = 200*.* (*b*) The model selection measures relative to the true model (for clarity, a table showing these values has been placed into electronic supplementary material, appendix O). (*c*) CPU time necessary to accurately estimate the model selection measures (within an uncertainty of 0.2) as a function of the number of observations *R*.

Figure 4*a* shows model selection measures for a subset of models characterized by *J*_sel_ (*κ_j_* = 1 for *j* ≤ *J*_sel_ and *κ_j_* = 0 for *j* > J_sel_). Because of the latent variables in the model, it now becomes necessary to consider the four definitions for DIC presented in equations (3.4) and (3.5). One immediate conclusion from figure 4*a* is the unreliability of DIC_6_ as a model selection measure. It has no minimum near to *J*_sel_ = 5 (the true model) and actually favours a model with no regressors at all. Similarly, DIC_4_ is also incorrect because it has a minimum at *J*_sel_ = 2.

Having discounted these two measures, we turn our attention to the other three curves in figure 4*a*. Owing to their large separation, they are difficult to compare on an absolute scale. Model comparison, however, only depends on the relative goodness of fit between models using the same measure. Consequently, figure 4*b* shows each remaining model selection measure relative to its value for the true model. The evidence-based results (solid red curve) exhibit a minimum at *J*_sel_ = 4, which is actually one less than the true value. However, one must also consider the certainty with which this optimum selection is made. A Bayes' factor of 10 represents strong evidence for one model over another, and this converts to a difference in −2 log(*P*(*y*)) of 4.6 between models. Therefore, because models with *J*_sel_ = 5 and 6 regressors are within 4.6 of *J*_sel_ = 4 in figure 4*b*, there is actually insufficient evidence to statistically differentiate between these possibilities (due to the limited size of the data).

The DIC_5_ measure in figure 4*b* follows the same general pattern as the evidence-based approach, but clearly approximations used in deriving DIC introduce a certain level of spurious fluctuation within this curve. While DIC_3_ correctly discounts models with too few regressors, it does not sufficiently penalize overfitting additional redundant parameters (which may be an artefact of not including the latent process likelihood in equation (3.4)).

Figure 4*c* shows the computational speeds of the various approaches. Here, the variance-based measures DIC_4_ and DIC_6_ are considerably slower (due to the difficulty in accurately estimating variances, as illustrated by the additional factor of $\sigma_{1}^{2}$ for DIC_2_ in equation (4.1)). The model evidence measure exhibits similar scaling properties here as when applied to the linear regression model.

### 5.3. Epidemiological models

**Figure 5.**
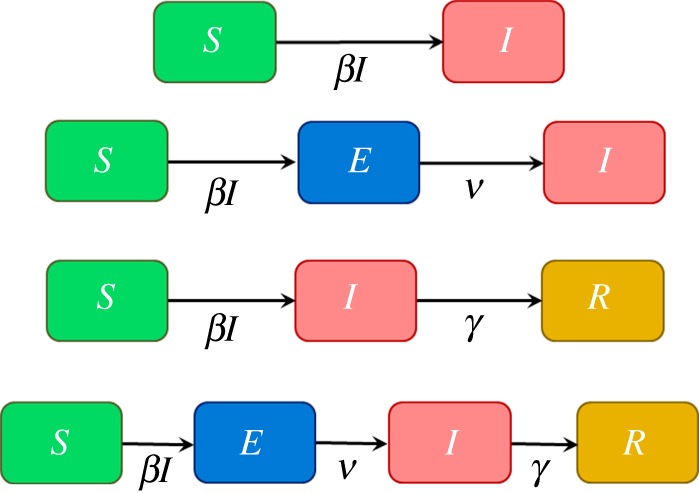
Four different models to describe disease dynamics. Compartments *S*, *E*, *I* and *R* refer to individuals being susceptible, exposed, infectious or recovered, respectively. The arrows indicate transition rates in disease status. (Note, these models are individual-based, so transitions from susceptible to infectious are given on a *per capita* basis *βI* rather than the usual *βSI*.)

**Figure 6.**
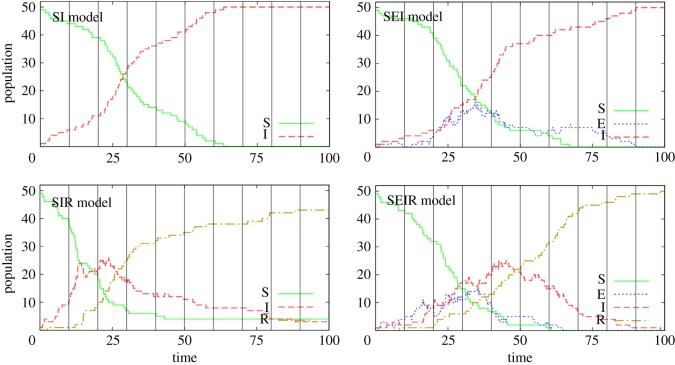
Simulated results from the four models presented in figure 5 (see electronic supplementary material, appendix M for details on how these are generated). Here, we consider a population that initially has 49 susceptible individuals and a single infected. The vertical black lines indicate times at which diagnostic tests are performed. (SI: *β* = 0.002, SEI: *β* = 0.003, *ν* = 0.1, SIR: *β* = 0.004, *γ* = 0.05, SEIR: *β* = 0.004, *ν* = 0.1, *γ* = 0.05.)

We now consider epidemiological models that have a non-MVN latent space. In particular, we consider four commonly used models for disease spread, as illustrated in figure 5. These models are suitable for homogeneously mixing populations but can readily be extended to account for spatial or social structure and other heterogeneities (e.g. [29–31]) and have been used widely to infer the characteristics of disease dynamics and spread. Individuals in the population are classified according to their disease status: *S* represents susceptible, *E* is exposed, *I* is infectious and *R* is recovered. The acronyms used to refer to the models in figure 5 correspond to which of these classifications the model contains (so, in order, these models are SI, SEI, SIR and SEIR, respectively). Model parameters *β, ν* and *γ* determine transition rates between these states. Consider a population of *p* = 50 individuals, 49 of whom are initially susceptible and one infected. Figure 6 shows the dynamic variation in the populations within *S*, *E*, *I* and *R* as a function of time when simulating from each of the four models in figure 5 (these simulations were performed using the Doob–Gillespie algorithm [32], as outlined in electronic supplementary material, appendix M).

Next, we consider observations made on the epidemic as it progresses. We assume that periodically all individuals in the population are tested to help identify their disease status. Typically, such diagnostic tests are imperfect, so we define a sensitivity Se = 0.8 (the probability that a truly infected individual, i.e. in the *E* or *I* states, tests positive) and specificity Sp = 0.95 (the probability that a truly uninfected individual tests negative).

**Figure 7.**
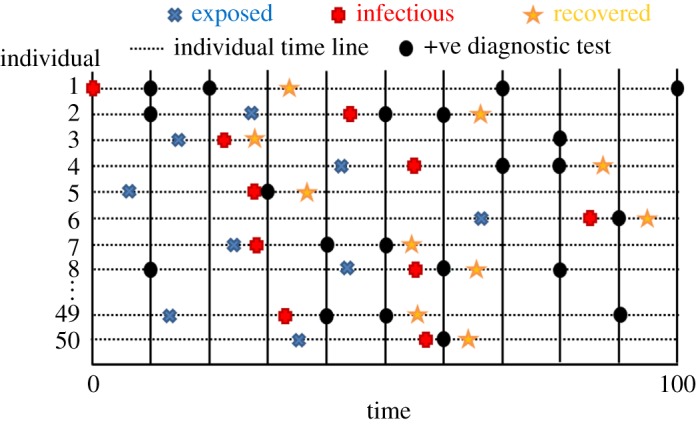
Representation of an individual-based SEIR compartmental model. The horizontal dotted lines represent timelines for individuals within the population, with blue crosses, red pluses and yellow stars denoting different event types (constituting the latent variables *x*). The vertical black lines are testing times, with positive test results indicated by black circles (note, occasionally positive test results are generated even when an individual is uninfected because the specificity is less than 1).

Figure 7 gives a diagrammatic representation of the system (here illustrated for the case of an SEIR model). The blue crosses, red pluses and yellow stars represent the times at which individuals become exposed, infectious or recover, respectively. Collectively, these ‘events’ *ξ* can be ordered on a single time line indexed by *e* (which runs from 1 to *E*).

The events themselves define the latent state *x* = {*ξ*}. The probability of generating this state, given a set of model parameters *θ* = {*β, ν, γ*}, is given by the latent process likelihood

5.7 $$\pi (x\;|\;\theta )=\prod_{e=1}^{E}\rho_{\xi_{e}}{\text{e}}^{-W(t_{e}-t_{e-1})},$$

where *t_e_* and *ξ_e_* are the time and type of event *e*.^13^ In the example of the SEIR model, event rates are given by

5.8 $$\rho_{\exp}=\beta I,\rho_{\inf}=\nu ,\rho_{\mathrm{rec}}=\gamma ,$$

(represented by the arrows in figure 5) and *W* = *Sρ*_exp_ + *Eρ*_inf_ + *Iρ*_rec_ is the total event rate (where *S*, *E* and *I* are the populations of susceptible, exposed and infected individuals immediately prior to time *t_e_*)_._ Note, these expressions are modified dependent on which model is being considered in figure 5.

Disease status testing times are indicated by the vertical lines in figure 7, and the black circles denote those animals which tested positive (otherwise negative). Collectively, these measurements represent the data *y*. The observed data likelihood is given by

5.9 $$P(y\;|\;\theta ,x)={\text{Se}}_{}^{N_{+\;|\;+}}{\left(1-\text{S}{\text{e}}_{}\right)}^{N_{-\;|\;+}}{\text{Sp}}_{}^{N_{-\;|\;-}}{\left(1-\text{Sp}\right)}^{N_{+\;|\;-}},$$

where *N_r_*_|*d*_ is the number of diagnostic tests which give result *r* from individuals with true disease status *d*.

**Figure 8.**
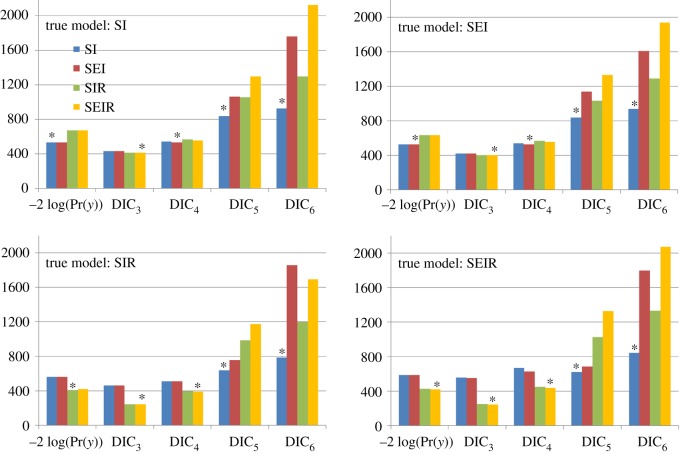
Model selection based on evidence and four DIC measures (equations (3.4) and (3.5)) applied to the simulated results from figure 6. Here, ‘true model’ refers to the model type used in simulating the data. Out of the potential model which could explain these data, the one with the smallest bar (with star on top) indicates the most likely model based on a given selection measure.

Based on the four simulated datasets shown in figure 6, we use model selection measures to attempt to uncover the true model and so reject the other possibilities. Results are shown in figure 8 (details of the MCMC proposals are given in electronic supplementary material, appendix N). Looking first at the evidence-based measure, we find that it selects the correct model in all cases, i.e. −2 log(*P*(*y*)) is always smallest (indicated by the star) for the true model. Contrast this situation with the DIC results in figure 8, where in many cases, the ‘best’ model is incorrect. In fact, all four DIC measures incorrectly identify the true model under at least one scenario.

**Figure 9.**
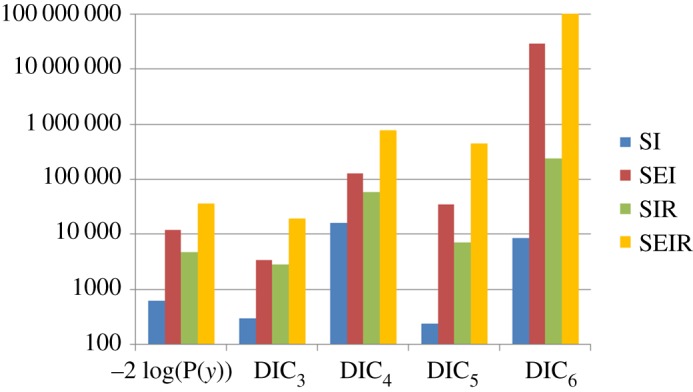
CPU time (in seconds) to accurately calculate the model selection measures (within an uncertainty of 0.2) for −2 log(*P*(*y*)) (using SS) and the DIC measures defined in equations (3.4) and (3.5).

Figure 9 gives a relative speed comparison between the methods. Again, we find that model evidence is no slower to calculate than the corresponding DIC measures (which, as discussed above, anyway represent poor criteria for identifying the data generating model).

## 6. Conclusion and discussion

This paper considers two different approaches which can be used to select the best model from a number of candidates: model evidence and DIC.

Model evidence was calculated using SS, which uses samples taken from multiple MCMC chains (run using inverse temperatures that bridge from the posterior to the prior) to construct an estimator. On the other hand, DIC is not uniquely defined and uses samples from a single posterior chain to generate a number of contrasting model selection measures.

The two approaches were compared using a number of benchmark problems (linear regression, mixed models and stochastic compartmental epidemic models). Model evidence was found to consistently predict the correct model. By contrast, the multiple definitions provided by DIC gave different and often contradictory results. While DIC has not been developed with model selection in mind (rather it is concerned with prediction accuracy of future datasets), this lack of consistency between measures (in some cases suggesting completely the wrong model) is of great concern.

Somewhat surprisingly, SS was found to take a similar amount of computational time as DIC, despite having to update a large number of chains. Furthermore, SS is inherently parallelizable, making further gains in speed using GPGPU technology relatively straightforward.

SS provides a robust and practical method for model selection applicable to a wide range of applications, e.g. for hierarchical mixed models used in the estimation of quantitative genetic effects [28] or phylogenetics [33]. It also has important implications for the application of statistical inference to Markov and semi-Markovian compartmental models widely used in epidemiology and ecology, where our results suggest that currently employed model selection approaches based on DIC measures [9–11] are likely to be misleading.

For very large systems estimating evidence using SS will inevitably become computationally demanding. With this in mind, model selection measures other than DIC are currently being developed which use single chain data to provide better model discrimination. One promising possibility is the widely applicable Bayesian information criterion (WBIC) [34]. This calculates the mean in the log of the observation probability at a specially chosen inverse temperature ϕ** *=* *1/log(*n*), where *n* is the sample size. It has been shown that while WBIC converges on the model evidence in the asymptotic limit [34], i.e. as *n *→ ∞, it can also produce biased results for small sample sizes and when priors are not very informative [35]. It will be interesting to see whether this or something else can be made sufficiently accurate and fast to become the new measure of choice.

## Supplementary Material

Appendices

## Supplementary Material

Illustrative code for linear regression model

## Supplementary Material

Illustrative code for mixed model

## Supplementary Material

Illustrative code for epidemiological models

## Footnotes

1 Typically, the components of the parameter vector *θ* that control the model dynamics are distinct from those that parametrize the observed data likelihood.

2 At least ‘practical’ when the number of models to choose among is relatively small, or when techniques such as sequential model selection are employed, which can miss the ‘best’ model.

3 To calculate model evidence, the prior must be proper ensuring that chain *K* remains bounded.

4 Here, burn-in and sampling are alternated as the inverse temperature jumps down in *K* steps starting at the posterior *ϕ*_1_ and ending up at the prior *ϕ_K_*.

5 Note, in the absence of latent variables, this quantity is often referred to as simply the ‘likelihood’, but for consistency within this paper, we persist in calling it the observed data likelihood.

6 A proper prior implies one that can be sampled from. An uninformative (flat) prior over an infinite parameter space is improper.

7 So an algorithm which uses *K* = 100 chains is no slower than the one which uses *K* = 50 chains. This remains true for larger and larger *K* until burning-in such a substantial number of chains becomes problematic.

8 A curious property of the graph in Figure 1*a* is that the gradient of the black line is equal to the variance in the log of the observed data likelihood.

9 This property also holds true when comparing DIC_4_ and DIC_6_ with DIC_3_ and DIC_5_ due to the relative inaccuracy of estimating variances compared to means.

10 It is worth noting that these stepwise methods are complicated when higher order, i.e. nonlinear, interactions in the explanatory variables are included, and under these circumstances, reversible jump MCMC may become the method of choice.

11 Note, a conjugate prior (instead of this flat prior) is often used to increase the speed at which the model evidence can be calculated. As this paper is focused on the generic performance of DIC and model evidence measures, we do not make use of this mathematical ‘trick’.

12 Here, four datasets are simulated and the CPU time using the procedure in electronic supplementary material, appendix I, are averaged over.

13 Here, we set *t*_0_ = 0 and, for convenience, assume an end ‘event’ *t_E_* = *t*_max_ with transition probability 1.

## Data accessibility

The findings of this paper are theoretical and do not rely on actual data. Exemplar code written in C++ to illustrate the operation of the various computational techniques is included in the electronic supplementary material.
