# Supplementary material for: Bayesian model evidence as a practical alternative to deviance information criterion
Source: R Soc Open Sci. 2018 Mar 21;5(3):171519. doi: 10.1098/rsos.171519 (PMC5882686; doi:10.1098/rsos.171519)
Supplement: Appendices [file rsos171519supp1.docx]

**Bayesian model evidence as a practical alternative to deviance information criterion** - appendices

C. M. Pooley1,2 and G. Marion2

1 The Roslin Institute, The University of Edinburgh, Midlothian, EH25 9RG, UK.

2 Biomathematics and Statistics Scotland, James Clerk Maxwell Building, The King's Buildings, Peter Guthrie Tait Road, Edinburgh, EH9 3FD UK.

Corresponding author: Dr C. M. Pooley

# Appendix A: Details of steppingstone sampling (SS)

### Theory

Equation (1.2) can be approximated by Monte Carlo integration through importance sampling [[1](#_ENREF_1)]. This is a general technique in which the expression within an integral is written in terms of two parts: (a) a normalised distribution which can be sampled from and (b) whatever remains. Successive samples are then taken from (a) and used to evaluated (b). The integral can then be approximated by averaging over these evaluations, with the estimate becoming increasingly accurate as the sample number is increased. In the case of Eq.(1.2), we imagine that *S* samples are taken from *π*(*x*|*θ*)*π*(*θ*)(which are typically generated by first drawing *θ* from the prior distribution *π*(*θ*) then simulating *x* from *π*(*x*|*θ*)) leading to the estimate

which converges on *P*(*y*) in the limit *S*→∞. Directly calculating *P*(*y*) using this approach is problematic for all but the simplest systems because the vast majority of samples *θs, xs* have a negligible observed data likelihood. This necessitates the use of more sophisticated techniques such as SS.

We define the shorthand *p=P*(*y*|*θ,x*) and introduce the probability density function *g(p)dp,* which represents the probability that a sample from the model has an observed data likelihood between *p* and *p+dp*. In the limit *S*→∞, Eq. can be rewritten as

$$P(y)=\int_{0}^{\infty} p\text{ }g(p)\text{ d}p.$$

Consider running *K* different MCMC chains with inverse temperatures *ϕ1=1* > *ϕ2* > … > *ϕK=0*. The probability density function for *p=P*(*y*|*θ,x*)in the *k-*th chain is taken to be *f­k(p)*. The Metropolis-Hastings condition in Eq.(2.1) ensures that states with higher observed data likelihood are preferentially selected compared to states sampled directly from the model. In particular, the relationship between *f­k* and *g* is given by

where *wk* are unknown normalisation factors. Considering chain *k=1*, substituting Eq. into Eq. and integrating leads to *P*(*y*)=*w1*. This can be written in the suggestive form

where *wK=1* follows from Eq. for *k=K* because *g* and *f­K* are both normalised. Next we establish how to estimate the separate ratios in Eq.. Multiplying Eq. byand equating *g* between adjacent chains gives

Multiplying this by, rearranging and integrating gives

MCMC from chain *k+1* produces *N* samples labelled$\theta_{i}^{k+1}$and$x_{i}^{k+1}$(where *i* goes from *1* up to a total *N*) which can be used to calculate$p=P(y|\theta_{i}^{k+1},x_{i}^{k+1})$. These represent correlated draws from the distribution *f­k+1*. Consequently, Monte Carlo integration through importance sampling can again be used to estimate the integral in Eq.

Substituting Eq. into Eq. yields the final estimator for the model evidence

where$\widehat{P}(y)\to P(y)$in the limit $N\to\infty$.

### Implementation

An “update” constitutes sequentially go over a complete set of possible proposals (*e.g.* in the simplest case we might go through each model parameter in turn and then each latent variable in turn) and accepting or rejecting these using the Metropolis-Hastings probability in Eq.(2.1). The SS algorithm can be described as follows:

**STEPPINGSTONE SAMPLING ALGORITHM:**

**Initialization**: We set $\theta_{1}^{1},x_{1}^{1}$ to be some initial state at *i = 1* on chain *k=1* (typically this involves generating a sample from the model). This initial state is then updated *Ninit* times which constitutes a burn-in phase. During this phase proposal distributions are optimally tuned (see Appendix B). The final state on chain *k=1* is then used as the initial state for chain *k=2,* which then undergoes a burn-in period itself. This process is repeated for all *K* chains. The following two steps are then iterated *N* times:

**Step 1:** Update chains *1* to *K-1.* For chain *K* directly sample from the model (if this is possible to do, otherwise it can also be updated).

**Step 2:** Swap states between chains (see Appendix C for details).

**Output:** Use Eq.(2.3) to estimate the model evidence.

# Appendix B: Tuning proposal parameters

The proposals used in this paper separately make changes to each individual parameter within the model. This is the simplest approach to take (although not necessarily the best, because potential correlations between parameters are ignored which can lead to poor mixing). Suppose *θj* is one parameter within *θ.* A new proposed value *θj,p* on chain *k* is sampled from the normal distribution

$$\theta_{j,p}\text{ \textasciitilde}N(\theta_{j,i}^{k},\lambda_{k,j}^{2})$$

centred at the parameter’s current value on the chain$\theta_{j,i}^{k}$with standard deviation *λk,j*. Tuning *λk,j* is important. If it is too large, very few proposals will be accepted and if too small, mixing will be slow. Motivated by adaptive MCMC [[2](#_ENREF_2), [3](#_ENREF_3)], a robust heuristic method for optimising *λk,j* within the burn-in period is as follows (note, after burn-in the proposal distributions are fixed to ensure that detailed balance is strictly satisfied). Initially, *λk,j* is set to a small quantity. Each time a proposed change on parameter *j* is accepted *λk,j* is updated according to

$$\lambda_{k,j}^{new}=\lambda_{k,j}\times1.01,$$

and when rejected

$$\lambda_{j}^{new}=\lambda_{k,j}\times0.995.$$

These numerical factors are chosen for two reasons: Firstly, the updates in Eqs. and balance each other out when acceptance occurs around 33% of the time, leading to a steady state solution for *λk,j*. Secondly, they are chosen to be sufficiently close to *1* to prevent large fluctuations in *λk,j* but sufficiently far to allow the steady state solution to be found within the burn-in period.

# Appendix C: Swapping between chains

One way to improving mixing when running multi-temperature MCMC is to perform swapping of states between adjacent chains. This works in the following way. From Eq.(2.2) the probability for sample *i* in chain *k* having a particular state$\theta_{i}^{k},x_{i}^{k}$is proportional to

where we use the shorthand $p_{k,i}=P(y|\theta_{i}^{k},x_{i}^{k})$. Taking all chains simultaneously, the joint probability is proportional to

We now consider swapping states between two adjacent chains *ks* and *k­s+1*. The total probability for this proposed state is proportional to

The corresponding Metropolis-Hastings probability is given by

Step 2 of the algorithm in appendix A is implemented by looping from *k­s=1 to ks*=*K-1* and swapping states $\theta_{i}^{k_{s}+1},x_{i}^{k_{s}+1}$ and$\theta_{i}^{k_{s}},x_{i}^{k_{s}}$with the probability given in Eq..

Swapping is generally computationally fast and almost always worth doing. It is of particular value to improve the mixing of chains with high temperature (low *ϕ*). This is because the chain *ϕK*=0 typically samples directly from the model (*i.e.* it does not suffer from correlations between successive samples that are usually problematic for efficient MCMC) and so swapping these uncorrelated states into the higher temperature chains is especially beneficial.

# Appendix D: Model selection measures given MVN distributions

Multiplying Eqs.(13) and (14) gives

$$P(y|\theta)\pi(\theta)=P(y|\left\langle\theta\right\rangle)e^{-\frac{1}{2}\left(\theta-\left\langle\theta\right\rangle\right)^{T}\Sigma^{-1}\left(\theta-\left\langle\theta\right\rangle\right)}\frac{e^{-\frac{1}{2}\left(\theta-\overline{\theta}\right)^{T}\Omega^{-1}\left(\theta-\overline{\theta}\right)}}{\left(2\pi\right)^{\frac{d}{2}}\sqrt{\left|\Omega\right|}}.$$

In anticipation of this being MVN we write

$$P(y|\theta)\pi(\theta)=C\frac{e^{-\frac{1}{2}\left(\theta-\mu\right)^{T}\psi\left(\theta-\mu\right)}}{\left(2\pi\right)^{\frac{d}{2}}\sqrt{\left|\psi\right|}},$$

where the constant *C,* mean *μ* and covariance matrix *ψ* need to be established. Equating Eqs. and leads to

$$\begin{matrix} P(y|\theta)\pi(\theta)=P(y|\left\langle\theta\right\rangle)e^{-\frac{1}{2}\left(\theta-\left\langle\theta\right\rangle\right)^{T}\Sigma^{-1}\left(\theta-\left\langle\theta\right\rangle\right)}\frac{e^{-\frac{1}{2}\left(\theta-\overline{\theta}\right)^{T}\Omega^{-1}\left(\theta-\theta\right)}}{\left(2\pi\right)^{\frac{d}{2}}\sqrt{\left|\Omega\right|}} \\ \text{ }=P(y|\left\langle\theta\right\rangle)\frac{e^{-\frac{1}{2}\theta^{T}\left(\Sigma^{-1}+\Omega^{-1}\right)\theta+\frac{1}{2}\left(\left\langle\theta\right\rangle^{T}\Sigma^{-1}+\overline{\theta}^{T}\Omega^{-1}\right)\theta+\frac{1}{2}\theta^{T}\left(\Sigma^{-1}\left\langle\theta\right\rangle+\Omega^{-1}\overline{\theta}\right)-\frac{1}{2}\left(\left\langle\theta\right\rangle^{T}\Sigma^{-1}\left\langle\theta\right\rangle+\overline{\theta}^{T}\Omega^{-1}\overline{\theta}\right)}}{\left(2\pi\right)^{\frac{d}{2}}\sqrt{\left|\Omega\right|}} \\ \text{ }=C\frac{e^{-\frac{1}{2}\theta^{T}\psi^{-1}\theta+\frac{1}{2}\mu^{T}\psi^{-1}\theta+\frac{1}{2}\theta^{T}\psi^{-1}\mu-\frac{1}{2}\mu^{T}\psi^{-1}\mu}}{\left(2\pi\right)^{\frac{d}{2}}\sqrt{\left|\psi\right|}}. \end{matrix}$$

Comparing terms on the second and third lines reveals the following relationships:

Substituting these results into Eq. gives

This expression can be integrated over parameter space (which effectively sets the square bracket to one) to give the final result in Eq.(3.8).

The expressions in Eq.(3.10) can be derived in the following way. Taking the log of the observed data likelihood in Eq.(3.6) gives

where ⟨…⟩ denotes an average over the posterior from Eq., Δ*θ=μ-⟨θ⟩* is the difference in mean parameters between the posterior and observed data likelihood and *δθ=θ-μ* represents the parameters relative to the posterior mean. Explicitly writing indices on matrices and using results such as ⟨*δθi*⟩=0 and ⟨*δθi δθj*⟩=*ψij* leads to

Substituting this into the expression for DIC1 in Eq.(3.2) leads to the analytical expression in Eq.(3.10).

The posterior variance in the observed data likelihood is given by

$$\begin{matrix} \mathrm{var}\left[\log\left(P(y|\theta)\right)\right]=\frac{1}{4}\left\langle\left[\left(\theta-\left\langle\theta\right\rangle\right)^{T}\Sigma^{-1}\left(\theta-\left\langle\theta\right\rangle\right)-\left(\text{Tr}(\Sigma^{-1}\psi)+\Delta\theta^{T}\Sigma^{-1}\Delta\theta\right)\right]^{2}\right\rangle\\ \text{ }=\frac{1}{4}\left\langle\left[\left(\delta\theta+\Delta\theta\right)^{T}\Sigma^{-1}\left(\delta\theta+\Delta\theta\right)-\left(\text{Tr}(\Sigma^{-1}\psi)+\Delta\theta^{T}\Sigma^{-1}\Delta\theta\right)\right]^{2}\right\rangle. \end{matrix}$$

Using the results ⟨*δθi δθj δθk* ⟩=0 and ⟨*δθi δθj δθk δθl*⟩=*ψijψkl* + *ψikψjl* + *ψilψjk* leads to

Combining the results from Eqs. and and the expression for DIC2 in Eq.(3.3) gives the analytical expression in Eq.(3.10).

# Appendix E: Estimating computational efficiency

This appendix derives the number of MCMC updates *U* (where one “update” refers to performing Metropolis-Hastings proposals on each of the model parameters and latent variables) to estimate the model selection measures to within an uncertainty of *ε*.

**SS:** We first obtain the sampling error when the model evidence is estimated using Eq.(2.3). Taking the log of this expression gives the estimate

where

and$l_{k,i}=\log\left(P(y|\theta_{i}^{k},x_{i}^{k})\right)$is the log of the observed data likelihood for the *i*-th sample from chain *k*.

Equation can be re-expressed as

where$\left\langle l_{k}\right\rangle$represents the average of $l_{k,i}$over a large number of MCMC iterations. Performing the Taylor series expansion

and truncating up to the first two terms (which is valid in the limit when *K* is large because *ϕk-1*- *ϕk* becomes small) leads to Eq. being approximated by

In the limit of large *N*, the MCMC based estimate for the average becomes closer and closer to its true value$\left\langle l_{k}\right\rangle$. Consequently, the second term within the log expression on the RHS of Eq. becomes smaller and smaller. Using the fact that log(1+*x*)≈*x* for small *x*, Eq. can be written

The first term on the RHS is exact (and on substitution into Eq. would lead to the exact value for the model evidence). The second term, on the other hand, comes from noised associated with MCMC sampling. An estimate for the variance of this noise is given by

When *i*=*i’*, the expectation on the RHS is simply given by the variance in$l_{k,i}$, which we denote$\sigma_{k}^{2}$. On the other hand, when *i*≠*i’* the expectation is still non-zero due to correlations in$l_{k,i}$along the MCMC chain. Taking the limit in which *N*→∞ (and using the concept of effective sample size as explained in appendix F), Eq. can be written

where

$$n_{k}^{cor}=1+2\sum_{\tau=1}^{\infty} F_{\tau},$$

represents a characteristic number of samples over which MCMC becomes uncorrelated and *Fτ* is the autocorrelation function for *l­k,i* (see appendix F for a definition).

Summing up the variances for the individual chains in Eq. leads to

*N* updates on *K* chains requires *U=NK* updates in all. Since the model selection measure is given by -2log(*P*(*y*)), equating 4 times Eq. with the desired variance ε2 leads to

**DIC1**: The term that dominates the sampling error in Eq.(3.2) is ⟨log(*P*(*y*|*θ*))⟩. When running the MCMC algorithm this average is estimated from

The variance in this quantity is given by

Remembering that DIC­1 contains a contribution of -4⟨log(*P*(*y*|*θ*))⟩ and that *U*=*N* because only one chain is used, we finally obtain

$$U=\frac{16n_{1}^{cor}\sigma_{1}^{2}}{\varepsilon^{2}}\text{ .}$$

**DIC2**: For large$\sigma_{1}$the term which dominates the uncertainty in Eq.(10) is the variance var[log(*P*(*y*|*θ*))]. Within MCMC this is estimated using

The expected variance in this quantity is given by

Following the same arguments as for Eq., this can be expressed as

where correlations in the MCMC are accounted for by a new quantity

$$n'_{k}^{cor}=1+2\sum_{\tau=1}^{\infty} F'_{\tau}$$

which uses $F'_{\tau}$, the autocorrelation function for$\left(l_{1,i}-\left\langle l_{1}\right\rangle\right)^{2}$(instead of simply $l_{1,i}$).

Assuming the observed data likelihood is approximately log-normally distributed, Eq. simplifies to

$$\mathrm{var}\left[\widehat{\mathrm{var}\left[\log\left(P(y|\theta)\right)\right]}\right]=\frac{2n'_{k}^{cor}\sigma_{1}^{4}}{N}.$$

Consequently the number of updates needed to calculate DIC2 to within an uncertainty of *ε* is

**
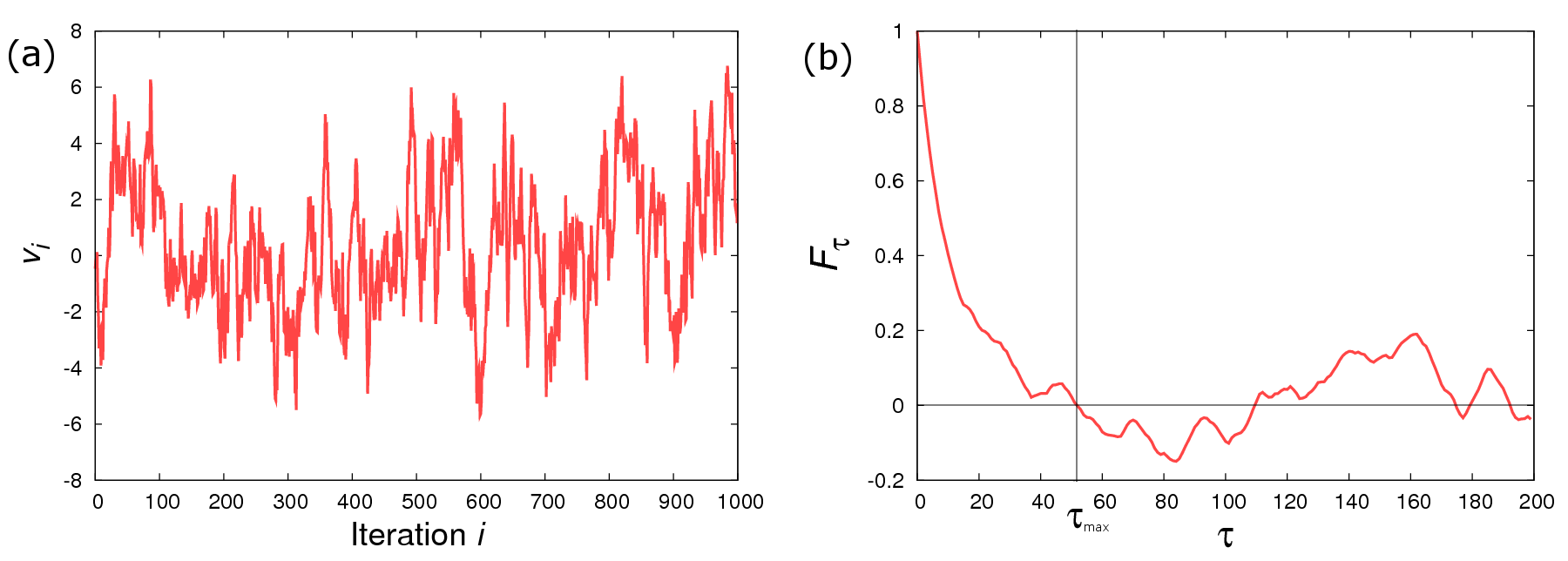
**

**Figure F:** (a) A typical trace plot and (b) a typical autocorrelation function.

# Appendix F: Effective sample number

Given *N* correlated MCMC samples of some quantity *υi* (where in this paper the focus is on the log of the observed data likelihood$v_{i}=\log(y|\theta_{i}^{k},x_{i}^{k})$) the autocorrelation function can be approximated using

$$F_{\tau}=\frac{1}{(N-\tau)\widehat{\sigma_{\nu}^{2}}}\sum_{i=1}^{N-\tau} \left(\nu_{i}-\widehat{\left\langle\nu\right\rangle}\right)\left(\nu_{i+\tau}-\widehat{\left\langle\nu\right\rangle}\right),$$

where estimates for the average and variance of *ν* are given by

The effective sample size is given by the actual sample number *N*, correcting for correlations between successive samples:

$$N_{k}^{eff}=\frac{N}{1+2\sum_{\tau=1}^{\infty} F_{\tau}}.$$

Correspondingly, we define the characteristic number of samples$n_{k}^{cor}$over which MCMC becomes uncorrelated is given by

$$n_{k}^{cor}=1+2\sum_{\tau=1}^{\infty} F_{\tau}.$$

When calculating$N_{k}^{eff}$or$n_{k}^{eff}$based on actual MCMC results, however, clearly the sums in Eqs. and cannot go to infinity. We now discuss how these quantities can be estimated in practice. Figure F shows a typical trace plot along with the corresponding autocorrelation function. Note, this function exhibits considerable fluctuations for large *τ*, and these can generate unwanted bias. The simplest way to deal with them is to truncate the sums in Eqs. and up to a maximum size *τ*max, which is defined to be the largest even value of *τ* for which the following condition holds[[1]](#footnote-1) (see [[4](#_ENREF_4)]):

$$F_{\tau}+F_{\tau+1}>0.$$

Essentially this represents the point at which the autocorrelation function intersects the *x*-axis, as illustrated by the vertical line in Fig. F(b).

# Appendix G: Evidence-based model selection using MCMC

In cases in which the number of potential models is large, one possibility to tackle model selection is through applying an MCMC scheme over model space (see, for example, [[5](#_ENREF_5)]). This makes use of the model evidence (which itself is calculated using a separate MCMC scheme such as SS) as the basis for jumping from one model to another (see algorithm below). Note, this is in contrast to reversible-jump MCMC that relies on a single MCMC scheme containing all models and specially designed proposals for jumping between models (which in practice can often be hard to implement[[2]](#footnote-2)).

The following example is for the linear regression model in section 5.1:

**MODEL SELECTION FOR LINEAR REGRESSION MODEL:**

**Initialisation:** Set index *i*=*1* and start with a model which incorporates all regressors, *i.e.* $\kappa_{j}^{i}=1$. Calculate an estimate of the model evidence$\widehat{P}_{i}$using SS.

**Step 1:** Randomly select a regressor *j­s* and flip its status to form a proposed state (*i.e.* $\kappa_{j_{s}}^{p}=1-\kappa_{j_{s}}^{i}$and$\kappa_{j}^{p}=\kappa_{j}^{i}$for *j*≠*js*).

**Step 2:** Calculate an estimate of the model evidence $\widehat{P}_{p}$for the proposed model.

**Step 3**: Accept the proposal with a Metropolis-Hastings probability

$$\max\left\{\frac{\widehat{P}_{p}}{\widehat{P}_{i}}\frac{\pi(\kappa^{p})}{\pi(\kappa^{i})},1\right\}$$

(*π*(*κ*) is the prior probability for a model defined by vector *κ*) and set $\widehat{P}_{i+1}=\widehat{P}_{p}$, *κi+1*= *κp* else set$\widehat{P}_{i+1}=\widehat{P}_{i}$, *κi+1*= *κi*.

**Step 4:** Increment *i* by *1* and jump to step *1*.

The validity of this algorithm rests on the fact that SS generates an unbiased estimate of the true model evidence (see [[6](#_ENREF_6)] for a discussion of this).

# Appendix H: DIC-based model selection using stepwise regression

Here the idea is to start at a particular model and make a succession of stepwise changes which aim to minimise the model selection measure [[7](#_ENREF_7)], in this case DIC. Implementation can be achieved in one of three ways:

**Forward selection** - Start with *κj*=*0* for *j*=*1*…*J’* and calculate the DIC. Go though each *j* in turn and calculate the DICs for new models for which *κj*=*1*. Select which of these changes *js* produces the greatest reduction in DIC and permanently set *κjs*=*1*. Repeat this procedure until no further reduction in DIC is possible.

**Backward elimination** - Start with *κj*=*1* for *j*=*1*…*J’* and calculate the DIC. Go though each *j* in turn and calculate the DICs for new models for which *κj*=*0*. Select which of these changes *js* produces the greatest reduction in DIC and permanently set *κjs*=*0*. Repeat this procedure until no further reduction in DIC is possible.

**Bidirectional elimination** – A combination of the two approach above which allows for both addition (*i.e. κjs*=*1*) an removal (*i.e.* *κjs*=*0*) of regressors.

# Appendix I: Calculating CPU time

**
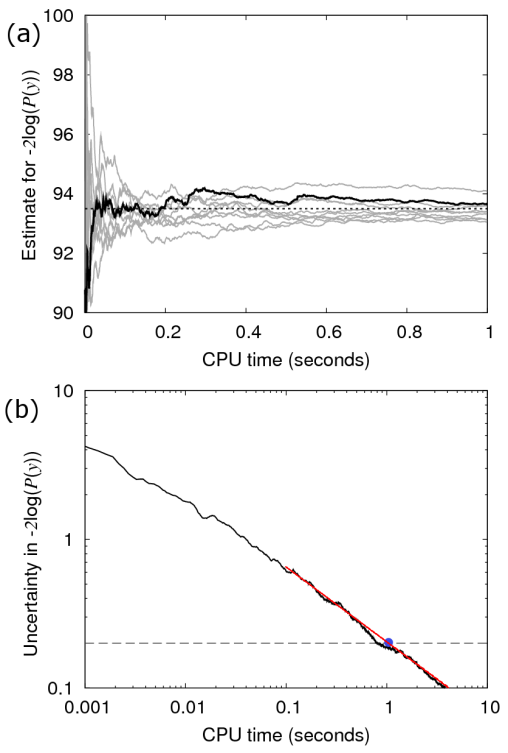
**

**Figure I:** (a) Shows estimates for -*2*log(*P*(*y*)) as a function of CPU time for a linear regression model with *J­sel=5* regressors. (b) The uncertainty in the approximations for -*2*log(*P*(*y*)) across *100* independent runs.

Here we outline a procedure for calculating the CPU time needed to estimate a model selection measure within an uncertainty of *0.2*. For illustrative purposes we consider SS for estimating -*2*log(*P*(*y*)), but the same basic method is also used for the DIC measures.

As the number of MCMC iterations *N* increases in Eq.(2.3) the more accurate the estimate for *P*(*y*) becomes. The black line in Fig. I(a) shows that -*2*log(*P*(*y*)) converges to the true solution (horizontal dashed line) as a function of computational time. In isolation, this curve cannot be used to estimate the time taken to reach a certain degree of uncertainty. Instead, we consider running the algorithm multiple times (*100* in total), with some of these different runs illustrated by the grey curves in Fig. I(a). The standard deviation between these runs can then be used to estimate the uncertainty in the estimate for -*2*log(*P*(*y*)) as a function of CPU time. An example of this is shown in Fig. I(b).

MCMC is a sampling procedure, so uncertainty in the model selection measure is expected to scale as the square root of the number of samples. Because Fig. I(b) is a log-log plot, such a scaling is represented by a line of slope -½ (solid red line), which can be fitted to the data. The point where this line intersects the desired uncertainty of *0.2* (blue filled circle) is used as the estimate for CPU time. To generate the figures in the results section, four data sets were first simulated and the average CPU time for inference was calculated (with 2×*103* burn-in updates and *N=105* sampling updates for SSand *N=106* sampling updates for DIC).

# Appendix J: Details of MCMC for the linear regression model

The following sets of proposals are used to perform a single “update” (note the explicit chain index *k* is omitted for clarity).

**UPDATE**

**Regressors** – Each regressor *βj* is considered in turn (where *j* runs from *1* to *Jsel*) and a new value is proposed from the normal distribution

$$\beta_{j,\mathrm{p}}\text{ \textasciitilde}N(\beta_{j,i},\lambda_{\beta_{j}}^{2})$$

centred at *βj,i* (the current value for*βj* on the chain being updated) with variance$\lambda_{\beta_{j}}^{2}$.

**Residual Variance** – A new value is proposed according to

$$\eta_{p}^{2}\text{ \textasciitilde}N(\eta_{i}^{2},\lambda_{\eta^{2}}^{2}).$$

The parameters$\lambda_{\beta_{j}}^{2}$and$\lambda_{\eta^{2}}^{2}$are automatically tuned during burn-in to give an average acceptance probability of approximately 33% (see appendix B for details).

In the case of DIC, updates are sequentially performed on the posterior chain and the proposals are accepted with the usual Metropolis-Hastings probability

$$\left\{\frac{P(y|\theta_{p},x_{p})\pi(x_{p}|\theta_{p})\pi(\theta_{p})}{P(y|\theta_{i},x_{i})\pi(x_{i}|\theta_{i})\pi(\theta_{i})}\frac{j_{p\to i}}{j_{i\to p}},1\right\},$$

where$\theta_{i},x_{i}$are the current model parameters and latent variables and$\theta_{p},x_{p}$are the proposed set.

In the case of SS each chain *k* is updated sequentially, but this time proposals are accepted with a modified Metropolis-Hastings probability

which explicitly includes *k* and incorporates the chain’s inverse temperature *ϕk*.

To generate the results in the paper 2×*103* burn-in updates were followed by *N=105* sampling updates in the case of SSand *N=106* sampling updates in the case of DIC.

# Appendix K: Calculating the relationship matrix A

To generate the population we first consider *P=50* individuals in the founder population. We then go through each of the *50* individuals in the 1st generation and randomly select their sire and dam (*s­r* and *d­r*) from the founder population (avoiding cases in which sire and dam are identical). This process is repeated for the 2nd and 3rd generations to make up a randomly mated population.

Calculation of the inverse relationship matrix **A**-1 is actually easier than calculating **A** (and considerable computationally faster for large matrixes because it is sparse). The following procedure is used:

1. Set all element of$\mathbf{A}^{-1}$to zero.
2. Loop over founder population members *r* and increment$\text{A}_{rr}^{-1}$.
3. Loop over non-founder population members *r* and add *2* to$\text{A}_{rr}^{-1}$, *-1* to$\text{A}_{s_{r}r}^{-1},\text{A}_{rs_{r}}^{-1},\text{A}_{d_{r}r}^{-1},\text{A}_{rd_{r}}^{-1}$and ½ to $\text{A}_{s_{r}s_{r}}^{-1},\text{A}_{d_{r}d_{r}}^{-1},\text{A}_{d_{r}s_{r}}^{-1},\text{A}_{s_{r}d_{r}}^{-1}$.

Because the relationship matrix is constant it becomes unnecessary to calculate the determinant of **A** in Eq.(5.6), as it cancels out when calculating Metropolis-Hastings acceptance probabilities.

# Appendix L: Details of the MCMC for the mixed model

The following sets of proposals are used to perform a single update (note the explicit chain index *k* is omitted for clarity).

**UPDATE**

**Regressors** – Each regressor *βj* is considered in turn (where *j* runs from *1* to *Jsel*) and a new value is proposed from the normal distribution

$$\beta_{j,p}\text{ \textasciitilde}N(\beta_{j,i},\lambda_{\beta_{j}}^{2})$$

centred at *βj,i* (the current value for*βj* on the chain being updated) with variance$\lambda_{\beta_{j}}^{2}$.

**Variances** – A new value for the environmental variance is proposed according to

$$\eta_{p}^{2}\text{ \textasciitilde}N(\eta_{i}^{2},\lambda_{\eta^{2}}^{2}),$$

and for the genetic variance

$$\omega_{p}^{2}\text{ \textasciitilde}N(\omega_{i}^{2},\lambda_{\omega^{2}}^{2}).$$

Note, we use here a simple Metropolis-Hastings random walk update for the variance components. Based on marginalisation of the posterior, it is possible to use Gibbs sampling for both of these quantities (as is usually done). However because of the prior distributions placed on *η*2 and *ω*2 (uniform between *0.1* and *2*) these Gibbs proposals frequently get rejected for high temperature chains, actually making this approach slower.

**Random effects** – When combining terms from the observed data likelihood in Eq.(5.5) and the latent process likelihood in Eq.(5.6), the posterior marginalised for a specific random effect *ur* is described by a normal distribution. Gibbs sampling from this distribution is achieved through

where matrix **C** is defined by

Here, **I** and **A** are the identity and relationship matrices, respectively. The notation **C***r,-r* denotes the row in matrix **C** excluding the element C*r,r*, and **u***-r,i* is a vector of random effects excluding *ur,i* for the current state *i*.

The parameters$\lambda_{\beta_{j}}^{2}$,$\lambda_{\eta^{2}}^{2}$and$\lambda_{\omega^{2}}^{2}$are automatically tuned during burn-in to give an acceptance probability of approximately 33% (see appendix B for details).

In the actual code some effort has been made to speed up proposals by pre-calculating computationally time consuming parts of the observed data likelihood and latent process likelihood, and then repeating certain types of proposal multiple times using a faster to calculate version of these quantities.

In the case of DIC, updates are sequentially performed on the posterior chain and the proposals are accepted with the usual Metropolis-Hastings probability

$$\left\{\frac{P(y|\theta_{p},x_{p})\pi(x_{p}|\theta_{p})\pi(\theta_{p})}{P(y|\theta_{i},x_{i})\pi(x_{i}|\theta_{i})\pi(\theta_{i})}\frac{j_{p\to i}}{j_{i\to p}},1\right\},$$

where$\theta_{i},x_{i}$are the current model parameters and latent variables and$\theta_{p},x_{p}$are the proposed set.

In the case of SS each chain *k* is updated sequentially, but this time proposals are accepted with a modified Metropolis-Hastings probability

which explicitly includes *k* and incorporates the chain’s inverse temperature *ϕk*.

To generate the results in the paper 2×*103* burn-in updates were followed by *N=105* sampling updates in the case of SSand *N=106* sampling updates in the case of DIC.

# Appendix M: The Doob-Gillespie algorithm

The Doob-Gillepie algorithm ([[8](#_ENREF_8)]) provides a way to simulate from discrete state-space continuous time Markov processes such as the compartmental models shown in Fig. 5. For illustrative purposes we focus on the SEIR model. The algorithm builds up a sequence of events in the following way:

**Initialization:** At time *t­0=0* all individuals are assumed to be susceptible apart from one that is taken to be infected. An index counting event number is set to *e=1*.

**Step 1**: Calculate the time to the next event. This is done by first calculating *W,* the total rate at which events (of any type) occur. Each individual *r* is classified as either being susceptible S, exposed E, infectious I or recovered R. The arrows in Fig. 5 denote transition rates between these states. An individual *r* classified as I, therefore, has a transition rate *ρ­r=γ* of recovering. The total event rate is found by summing over all individuals *W*=Σ­*rρr*­. Drawing a random inter-event time Δ*t* from the exponential distribution *We-W*Δ*t* is achieved by sampling a new event time in the following way

$$t_{e}=t_{e-1}-\frac{\log(u)}{R},$$

where *u* is a (uniform) randomly generated number between *0* and *1*.

If *te* is greater than the simulation time *tmax* the algorithm is terminated.

**Step 2**: Select which individual *q* undergoes a disease status transition with probability

$$\mathrm{Prob}(q=r)=\frac{\rho_{r}}{W}.$$

**Step 3**: Update the state of individual *q*.

**Step 4:** Increment *e* and jump to step 1.

# Appendix N: Details of the MCMC for epidemiological models

The following sets of proposals are used to perform a single update (note the explicit chain index *k* is omitted for clarity).

**UPDATE**

**Parameters** – The number of parameters is model dependent. For example the SI model only contains a single parameter *θ*={*β*}, whereas the SEIR model has three *θ*={*β,ν,γ*}. During the MCMC procedure we consider each parameter *j* in turn and propose a new value according to

$$\theta_{j,p}\text{ \textasciitilde}N(\theta_{j,i},\lambda_{j}^{2}).$$

**Move events** – An event *e* is randomly selected from the *E* events within *ξ*. A new event time is proposed according to

$$t_{p}\text{ \textasciitilde}N(t_{e,i},\lambda_{t}^{2}).$$

Note, this new time does not include a subscript *e* because typically there is a reordering of events under this transformation. If the move in inconsistent (*e.g.* a recovery event in placed before an infection event) the proposal is immediately rejected.

**Add Event** – An individual *r* is randomly selected from the population. The final event *e* that affects *r* is established (if no such event exists we set *e=0* to be the event at the beginning of the simulation). If that state is not terminal (*i.e.* there exists a transition from that state to another) then the transition is added to the time line at a point randomly selected between *te* and *tmax* (the maximum simulation time). The ratio of forward to reverse proposal probabilities in Eq.(2.1) is given by

$$\frac{j_{p\to i}}{j_{i\to p}}=t_{\max}-t_{e}.$$

**Remove Event** – An individual *r* is randomly selected from the population. The final event *e* that affects *r* is established (if no such event exists then no proposal is made). Event *e* is removed. In this new state the final event *e’* that affects *r* is established (if no such event exists then we set *e’=0* to be the event at the beginning of the simulation). In this case, the ratio of forward to reverse proposal probabilities in Eq.(2.1) is given by

$$\frac{j_{p\to i}}{j_{i\to p}}=\frac{1}{t_{\max}-t_{e'}}.$$

The parameters$\lambda_{j}^{2}$and$\lambda_{t}^{2}$are automatically tuned during burn-in to give an acceptance probability of approximately 33% (see appendix B for details).

A single “update” consists of twenty sets of proposals for each of the parameters (for which the likelihood calculation is sped up by using the fact that *ξ* is fixed) followed by five randomly selected move event proposals, and finally two randomly selected add/remove event proposals. This ensured an approximate 20/80 split in CPU time between updating model parameters and latent variables.

In the case of DIC, updates are sequentially performed on the posterior chain and the proposals are accepted with the usual Metropolis-Hastings probability

$$\left\{\frac{P(y|\theta_{p},x_{p})\pi(x_{p}|\theta_{p})\pi(\theta_{p})}{P(y|\theta_{i},x_{i})\pi(x_{i}|\theta_{i})\pi(\theta_{i})}\frac{j_{p\to i}}{j_{i\to p}},1\right\},$$

where$\theta_{i},x_{i}$are the current model parameters and latent variables and$\theta_{p},x_{p}$are the proposed set.

In the case of SS each chain *k* is updated sequentially, but this time proposals are accepted with a modified Metropolis-Hastings probability

which explicitly includes *k* and incorporates the chain’s inverse temperature *ϕk*.

To generate the results in the paper *104* burn-in updates were followed by *N=105* sampling updates in the case of SSand *N=106* sampling updates in the case of DIC. The prior for β were chosen to be flat and in the range 0.0004-0.01, for τ in the range 0.02-0.2, and for γ in the range 0.01-0.1.

# Appendix O: Table showing model selection measures

This table shows the model selection measures used in Fig. 4(a) and (b).

| *J­sel* | -2log(*P*(*y*)) | DIC3 | DIC5 |
| --- | --- | --- | --- |
| 0 | 327.4 | 254.0 | 485.9 |
| 1 | 303.7 | 276.3 | 337.8 |
| 2 | 296.6 | 265.8 | 327.8 |
| 3 | 286.3 | 248.1 | 316.5 |
| 4 | 278.9 - Selected | 226.6 | 322.4 |
| 5 – True model | 281.5 | 230.5 | 314.5 |
| 6 | 281.4 | 224.7 - Selected | 313.2 - Selected |
| 7 | 285.6 | 226.2 | 316.2 |
| 8 | 288.7 | 226.7 | 317.3 |
| 9 | 293.0 | 228.9 | 320.2 |
| 10 | 294.1 | 221.5 | 324.9 |

Note, the red entries show that whilst *Jsel*=4 is chosen to be the “best” model based on model evidence, those models with *Jsel*=5 and *Jsel*=6 are also candidates as they lie within 4.6 units of *Jsel*=4.

# References

[1] Evans, M. & Swartz, T. 2000 *Approximating integrals via Monte Carlo and deterministic methods*. Oxford ; New York, Oxford University Press; ix, 288 p. p.

[2] Andrieu, C. & Thoms, J. 2008 A tutorial on adaptive MCMC. *Stat Comput* **18**, 343-373.

[3] Roberts, G.O. & Rosenthal, J.S. 2009 Examples of Adaptive MCMC. *J Comput Graph Stat* **18**, 349-367.

[4] Geyer, C.J. 1992 Practical Markov Chain Monte Carlo. *Statist. Sci.* **7**, 473-483.

[5] Chipman, H., George, E.I. & McCulloch, R.E. 2001 The Practical Implementation of Bayesian Model Selection. In *Model selection* (ed. P. Lahiri), pp. 65-116. Beachwood, OH, Institute of Mathematical Statistics.

[6] Andrieu, C., Doucet, A. & Holenstein, R. 2010 Particle Markov chain Monte Carlo methods. *J. R. Stat. Soc. B* **72**, 269-342.

[7] Hocking, R.R. 1976 A Biometrics Invited Paper. The Analysis and Selection of Variables in Linear Regression. *Biometrics* **32**, 1-49.

[8] Gillespie, D.T. 1977 Exact Stochastic Simulation of Coupled Chemical-Reactions. *J. Phys. Chem.* **81**, 2340-2361.

1. The reason Eq. is not simply *Fτ*>0 is down to a curious quirk of autocorrelation functions generated from MCMC: in the limit of large sample size the sum of adjacent autocorrelation values is guaranteed to be positive , whereas individual values are not. [↑](#footnote-ref-1)
2. For example, adding an extra compartment into the epidemic models from section 5.3 means revising the event time lines for all the individuals. Ideally this would be achieved by sampling these new events from the posterior of the new model. Such sampling, however, is usually not possible (or sufficiently accurate) leading to negligibly small acceptance probabilities for switching between models. [↑](#footnote-ref-2)
